# PRF‐Assisted Crestal Sinus Floor Elevation With Bone Tap Drills in the Implant Surgical Kit (A Simplified Minimally Invasive Technique): A Case Series

**DOI:** 10.1002/ccr3.71506

**Published:** 2025-11-20

**Authors:** Mehdi Ekhlasmandkermani, Behzad Houshmand, Emir Ilkerli, Fatemeh Goudarzimoghaddam

**Affiliations:** ^1^ School of Dentistry Kerman University of Medical Sciences Kerman Iran; ^2^ Department of Periodontics, School of Dentistry Shahid Beheshti University of Medical Sciences Tehran Iran; ^3^ Department of Orthopedics and Traumatology Ostemed Klinik Bremervörde Bremervörde Germany; ^4^ Department of Periodontics, School of Dentistry Babol University of Medical Sciences Mazandaran Iran

**Keywords:** dental implant, oral surgery, platelet‐rich fibrin, sinus floor elevation

## Abstract

Here, we present two Persian 58‐ and 39‐year‐old females with an edentulous posterior maxilla in the first molar region and a minimum residual bone height (RBH) of 4 mm. Sinus floor elevation with crestal approach was considered. Platelet‐rich fibrin (PRF) was utilized as a crucial safeguard during sinus floor elevation with the assistance of bone tap drills featuring round ends. The placement of cylindrical implants was carried out simultaneously. Before applying this technique “in vivo” on human subjects, the “ex vivo” efficacy of PRF‐assisted crestal sinus floor elevation with bone tap drills was evaluated. It seems that PRF‐assisted crestal sinus floor elevation with bone tap drills can be used as a simplified minimally invasive technique.

**Summary**

- The present study provides evidence that sinus floor elevation up to 2–3 mm could be effectively accomplished by incorporating PRF and bone tap drills.
- The presented technique shows promising results in these specific clinical scenarios, though further controlled studies are needed to validate its efficacy.

## 1 Introduction

Ongoing maxillary sinus pneumatization and crestal bone loss are factors that can have a significant impact on the RBH. In general, the RBH value is the decisive factor in selecting between the lateral and crestal techniques. When the RBH is < 5 mm, it is recommended to employ a lateral approach utilizing grafting material [1].

The technique of the crestal approach, which was originally introduced by Tatum in 1986, underwent a significant modification by Summers in 1994 [2, 3]. This technique involves the utilization of an osteotome, which may induce complications for the patient as a result of enduring multiple blows [4, 5]. Subsequently, this approach has been gradually replaced by other methods that offer a safer alternative [6, 7]. In 2020, a modified technique known as VEST (Vertically Expander Screw Technique) was proposed to gradually separate the sinus membrane from the sinus floor bone. This innovative technique employs screws from bone expander kits to elevate the membrane upwards [8]. According to research conducted using endoscopic techniques to evaluate the risk of sinus membrane perforation, it has been demonstrated that sinus floor elevation with a crestal approach between 3 and 5 mm is associated with the lowest risk of perforation [9, 10]. Based on the findings of a recent systematic review, it has been suggested that dental implants with a shorter length (< 8 mm) may present a viable alternative to the standard implants of 10 mm or more in cases of residual bone height deficiency in the posterior maxilla [11]. Three factors may increase the possibility of membrane perforation. The first is the direct contact of the sinus floor elevation instrument with the membrane, which may occur if the force applied is not carefully controlled. Second, direct contact of the bone graft material with the sinus membrane and the associated risk of perforation, which stems from the irregularity of graft particles. Third, direct contact between the implant apex and the sinus membrane during implant insertion. Therefore, the implant's geometry plays a crucial role in preventing sinus membrane perforation.

In the context of the sinus floor elevation procedure, the utilization of flexible biomaterials such as PRF prior to device or implant insertion may potentially mitigate the incidence of membrane perforation. The presence of a fibrin network within the structure of PRF suggests that PRF may function similarly to a mucous adhesive. It is anticipated that PRF can effectively adhere to the schneiderian membrane, thereby providing coverage and protection against potential perforations [12]. After three decades of utilization of autogenous platelet concentrate, recent studies have highlighted the expanding role of these biomaterials not only in soft tissue repair but also in hard tissue repair.

This article presents a modified technique of VES, using the bone tap drill in the implant surgical kit available in the Straumann surgical kit, combined with PRF to safeguard the sinus membrane while simultaneously placing 8 mm implants.

## 2 Case Presentation

The study included 2 patients (2 women). Both participants in the study provided their informed consent and were fully aware of the treatment protocol before being admitted to the private clinic. Two systemically healthy and non‐smoker 58‐ and 39‐year‐old females with a complaint of edentulism of the upper right and left first molar, respectively (Figures 3A and 4A). The soft tissue presented without the need for enhancement regarding contour or mucosal attachment. Additionally, there was a minimum of 4 mm of residual sinus floor material, and the sinus was clear of any pathological lesions. Furthermore, all patients had sufficient bone width to facilitate the simultaneous placement of a standard diameter implant, eliminating the necessity for horizontal bone reconstruction.

## 3 Investigation and Treatment

**FIGURE 1:**
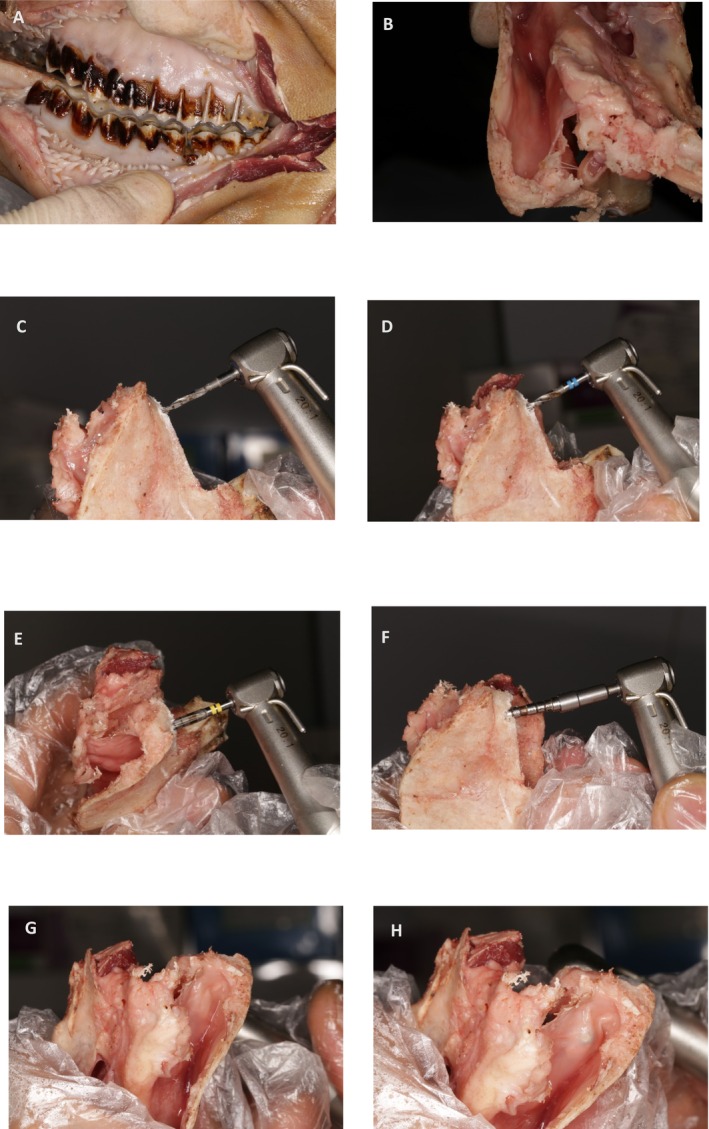
Sheep model. (A) A skin incision is made to access the posterior area of the maxilla. (B) Separation of the bone that contains the sinus from the maxilla. (C) Inserting the starter drill within the close distance of the sinus floor. (D) Inserting a drill with a diameter of 2.2 mm within the close distance of the sinus floor. (E) Inserting a 2.8‐mm‐diameter drill to perforate the cortical bone of the sinus floor. (F) A bone tap drill is used to enter the sinus cavity and elevate the schneiderian membrane. (G) Before insertion of the bone tap drill into the sinus cavity, that the sinus membrane was not elevated. (H) After insertion of the bone tap drill into the sinus cavity, it was observed that the sinus membrane was elevated without any perforation, as indicated by the arrow.

Before implementing this technique to elevate the sinus floor on patients, a thorough investigation was carried out on this surgical procedure for the maxillary sinus of sheep (Figure 1). The primary objective of this animal study is to assess the safety of the bone tap drill in direct contact with the sinus membrane. According to a study conducted in the year 2008, using fresh sheep heads can serve as an effective model for training in maxillary sinus floor elevation [13]. In this animal study, models were employed to establish the lateral window. To achieve adequate access to the vestibular depth, it is necessary to make an incision in the skin located in the corner of the mouth towards the posterior region (Figure 1A). To directly observe the response of the sinus membrane and elevate it from the crestal area, it is necessary to make a horizontal incision below the orbit. This incision offers a superior view of the interior of the sinus from a coronal perspective (Figure 1B). Following the extraction of the molar tooth from the sheep in the prepared samples, drilling was initiated following a predetermined sequence. The sinus membrane elevation from the crestal area with the aid of a bone tap drill denotes the gradual disengagement of the sinus membrane from the sinus floor bone (Figure 1C–F). After careful consideration, it has been determined that the bone tap drill technique is a safe approach for sinus floor elevation in patients who are eligible for a crestal approach (Figure 1G,H).

**FIGURE 2:**
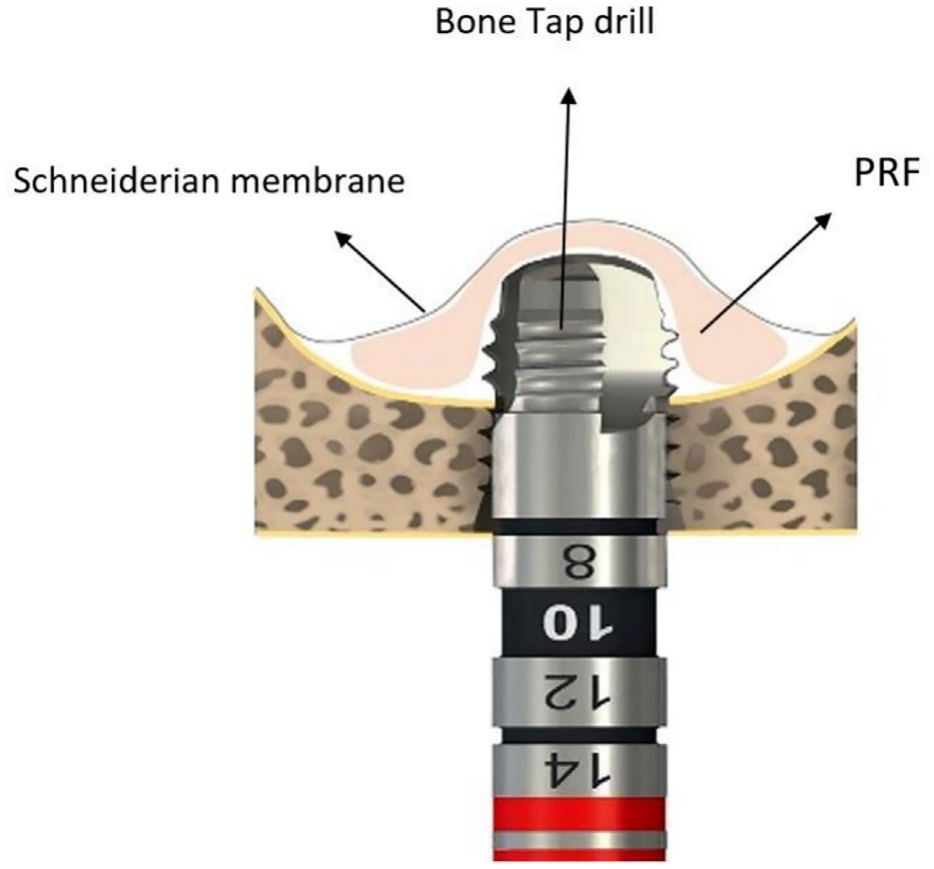
Schematic illustration of PRF transfer into the sinus cavity via bone tap drill. Utilizing a bone tap drill at a low speed to transfer PRF facilitates the flow of PRF beneath the sinus membrane, thereby minimizing direct contact between the drill and the membrane.

**FIGURE 3:**
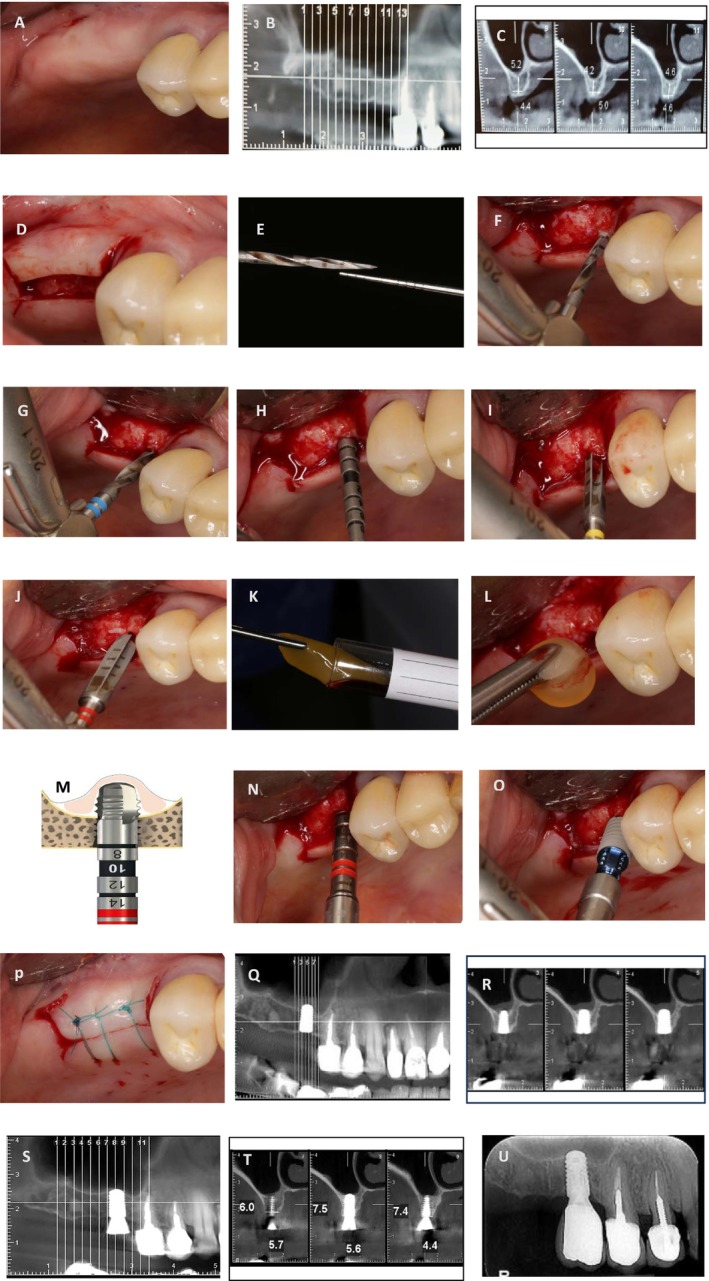
Case 1. (A) Pre‐operation view. (B, C) The ideal position of the implant. (D) Mid‐crestal incision. (E, F) The penetration depth of the starter drill within the close distance of the cortical bone of the sinus floor. (G) Insert a drill with a diameter of 2.2 mm, in the same manner as the starter drill. (H) Three‐dimensional positioning. (I) Inserting a 2.8‐mm‐diameter taper‐line drill based on RBH to perforate the cortical bone of the sinus floor. (J) Inserting a 3.5‐mm‐diameter taper‐line drill based on RBH to expand the cortical sinus floor hole. (K, L) Inserting PRF into the osteotomy hole. (M, N) The sinus membrane was elevated by transferring PRF using a taper bone tap drill into the sinus cavity. (O) Implant insertion (4.1 × 8 mm, BL). (P) Suturing (Nylon 5‐0). (Q, R) The CBCT view immediately following surgery. (S, T) The CBCT after 5 months. (U) The periapical view 1 year after the prosthetic delivery phase.

**FIGURE 4:**
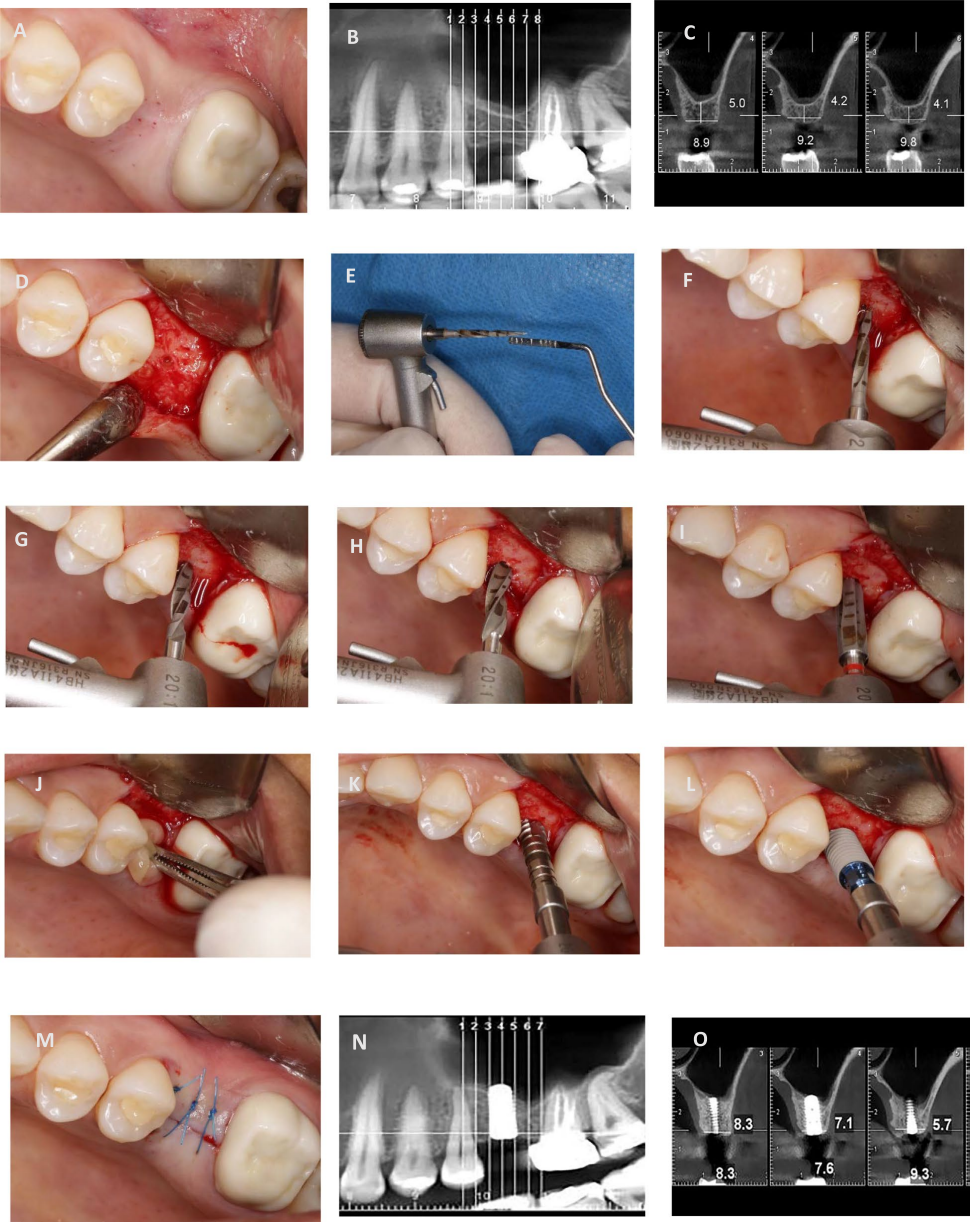
Case 2. (A) Pre‐operation view. (B, C) The ideal position of the implant. (D) Mid‐crestal incision. (E, F) Penetration rate of starter drill within the close distance of the cortical bone of the sinus floor. (G) Inserting a drill with a diameter of 2.2 mm, in the same manner as the starter drill. (H) Inserting a 2.8‐mm‐diameter drill based on RBH to perforate the cortical bone of the sinus floor. (I) Inserting a 3.5‐mm‐diameter taper‐line drill based on RBH to expand the sinus floor hole. (J) Inserting PRF into the osteotomy hole. (K) The sinus membrane was elevated by transferring PRF using a bone tap drill into the sinus cavity. (L) Implant insertion (4.1 × 8 mm, BL). (M) Suturing (Nylon 5‐0). (N, O) CBCT after 5 months.

To ensure consistent evaluations across both patients, Cone Beam Computed Tomography (CBCT) was requested. After clinical and paraclinical examination, simultaneous sinus floor elevation with implant placement (4.1 × 8 BL. Straumann. Switzerland) was considered. The results of the CBCT scan revealed that the most optimal positions for the implant from the most mesial and most distal points were 5.2 and 4.6 mm in Case 1 and 5 and 4.1 mm in Case 2, respectively (Figures 3B,C and 4B,C). Prior to the commencement of the surgical procedure, the patients were administered a 0.2% chlorhexidine mouthwash for 1 min. Additionally, the necessary amount of blood was drawn from the patients for the preparation of PRF. The collected blood was processed in the centrifuge (Silfradent medifuge. Italy) and placed in specialized red‐colored tubes to produce the required PRF membrane (Figure 3K). Following the administration of the anesthesia via the infiltration approach, a mid‐crestal incision was made on the edentulous ridge (Figures 3D and 4D). Based on the RBH in each case, the position of the osteotomy hole in proximity to the sinus floor was determined by employing a starter drill with a diameter of 1.6 mm (Figures 3E,F and 4E,F) and a subsequent drill with a diameter of 2.2 mm (Figures 3G and 4G). Following the verification of accurate three‐dimensional positioning of the implant using a parallel pin (Figure 3H), a 2.8 diameter drill was employed to perforate the sinus floor bone through the osteotomy hole without any penetration into the sinus cavity (Figures 3I and 4H). The third drill, with a diameter of 3.5 mm, was utilized in the same manner, solely to the extent of the perforation of the sinus floor cortical bone (Figures 3J and 4I). The drilling speed for preparation of the osteotomy hole and reaching the sinus floor is set at a maximum of 800 rpm, in accordance with the drilling protocol provided by the implant manufacturer (Straumann). Additionally, the speed for using the bone tap drill to transfer PRF and the subsequent implant placement adhered to the previously outlined protocol, with a speed of 15 rpm and a maximum torque setting of 35 N/cm. To ensure the primary stability of Straumann's cylindrical implants, in case of low bone density, under‐drilling is recommended using the final drill (3.5 mm‐diameter) selected from the taper line. During this stage, a portion of the PRF membrane is inserted into the osteotomy hole to safeguard the sinus membrane. In the subsequent step, the bone tap drill from the taper line (BLT, 4.1 mm‐diameter) in case of low bone density is used as a PRF carrier into the osteotomy hole (Figure 2). It is essential to ensure that the apex of the drill does not penetrate beyond 3 mm through the perforation of the sinus floor bone. Based on an experimental study, it has been observed that dental implants which penetrate more than 3 mm into the maxillary sinus and subsequently damage the sinus membrane can lead to an irreparable perforation in the apex of the implant [14]. Prior to the implant insertion, it is recommended that a supplementary amount of PRF be applied to the osteotomy hole. This will serve to prevent the implant apex from coming into direct contact with the sinus membrane (Figures 3K–O and Figure 4J–L). Finally, the flap was repositioned with sutures (Nylon 5‐0, SUPA, Iran) (Figures 3P and 4M). The surgical procedures for Cases 1 and 2 have been illustrated in Figures 3 and 4, respectively.

The administration of antibiotics was commenced a day before the surgical procedure with Co‐Amoxiclav 625 mg and was continued until one week following the surgery, at which point the sutures were removed. Ibuprofen was prescribed as a painkiller. Radiographs were obtained CBCT The CBCT view immediately following the surgical procedure for Case 1 is shown in Figure 3Q,R.

## 4 Result and Conclusion

There were no signs or symptoms of infection or treatment failure when we returned to the site. Additionally, due to the simplicity of the surgical procedure, the patients expressed a high level of satisfaction with the treatment.

In both patients, CBCT was acquired immediately before the second phase after 5 months of the healing period. In Case 1, the change in bone height observed was from 5.0 to 8.3 mm for the mesial implant and from 4.1 to 5.7 mm for the distal implant (Figure 3S,T). In Case 2, the measurements were from 5.2 to 7.5 mm and from 4.6 to 7.4 mm, respectively (Figure 4N,O). The periapical view 1 year after delivery of the prosthesis in Case 1 can be used to demonstrate the stability of the new bone around the implant (Figure 3U). The results of this comparison are summarized in Table 1.

**TABLE 1:** Summary of pre‐ and post‐operative parameters for the two cases.

Parameter	Case 1—Pre‐op	Case 1—5 months post‐op	Case 2—Pre‐op	Case 2—5 months post‐op
Residual bone height (RBH, mm)				
Mesial	4.6	7.4	5	8.3
Distal	5.2	7.5	4.1	5.7
Implant penetration depth (mm)				
Mesial	3.4	—	3	
Distal	2.8	—	3.9	
New bone height (mm)				
Mesial	—	2.8	—	3.3
Distal	—	2.3	—	1.6
Sinus membrane thickening	Not seen	Not seen	< 2 mm	< 1 mm
Sinus membrane perforation	No	—	No	—

## 5 Discussion

According to current recommendations, short implants (6–8 mm in length) are now considered a favorable alternative to standard implants exceeding 8 mm in length [11]. In cases where the RBH is < 6 mm, it may be beneficial to consider utilizing the crestal approach as an alternative to the lateral approach. Sometimes anatomical limitations, such as the presence of septa within the sinus or thick branches of arteries in the lateral wall, may preclude the use of a lateral surgical approach. When adequate bone is available, it is important to consider that the crestal approach may present fewer associated risks in these situations [15]. When the RBH is < 4 mm, the technical sensitivity associated with the crestal approach tends to increase, which in turn, may lead to a higher probability of sinus membrane perforation. According to a study, the factors that determine membrane perforation include RBH and the amount of vertical elevation height [16]. In cases where the RBH is 4–6 mm, the crestal approach is recommended. When dealing with an RBH < 4 mm, it is advisable to consider the lateral approach as the primary choice. The thickness of the sinus membrane is a significant factor influencing the risk of perforation. This issue is highlighted in the article by Testori et al., which indicates that the probability of perforation increases in cases where the membrane thickness is < 0.8 mm or exceeds 3 mm [17].

It is essential to prioritize the selection of cylindrical implants with a tapered end for close sinus lift procedures. This design minimizes the risk of damaging the Schneiderian membrane if the implant comes into direct contact with it. In the technique presented using the bone tap drill along with utilizing PRF, there has been an increase in the bone height from 5 to 8.3 in the mesial part of the implant, and from 4.1 to 5.7 in the distal part in the first instance. In the second one, the bone height has increased from 5.2 to 7.5 in the mesial part of the implant, and from 4.6 to 7.4 in the distal part. In both cases, there was no discernible bone formation beyond the implant apex.

In cases where the sinus floor elevation is minimal, the graftless approach may prove to be an advantageous option. A 2017 systematic review was conducted to analyze studies that implemented the graftless approach for sinus floor elevation. A meta‐analysis of participants was included in the study, with an average RBH of 5.7 ± 1.7 mm. After the healing period, the average vertical bone gain was found to be 3.8 ± 0.34 mm [18]. The present article outlines a technique that employs bone tap drills to increase height around the implant by 2–3 mm, which aligns with the outcomes reported in a previous systematic review. The application of PRF has been found to be effective in safeguarding the sinus membrane in cases where sinus perforation is not encountered during the preliminary stages of drilling. If the bone graft material is in direct contact with the sinus membrane, there is a possibility that micro‐perforations may gradually enlarge, potentially leading to the entry of the bone material into the sinus cavity [19]. Therefore, in certain limited cases of sinus floor elevation, the incorporation of PRF has the potential to eliminate the requirement of bone grafts in the crestal approach [20].

The findings of the recent technique indicate a lack of bone formation beyond the implant apex. The flexibility and low persistence of PRF render it incapable of preventing the collapse of the sinus membrane on the implant apex.

The findings presented in our article align with those of a recent retrospective study, indicating that new bone formation beyond the apex of the implant is not observed in the absence of bone material. This retrospective study revealed that the bone formation around the implants remains stable even after a 3‐year follow‐up. The study utilized PRF, with or without the addition of bone materials. Therefore, bone formation is expected to occur when employing bone material beyond the implant apex [21]. Following a comprehensive 1‐year follow‐up, we have found that the implant demonstrates sufficient stability within the fabricated bone to withstand the occlusal load.

The depth of implant penetration into the sinus, particularly in the absence of bone material, may increase the risk of sinus membrane perforation. An experimental study has demonstrated this correlation [22]. In this case report, 8 mm implants were selected to mitigate the penetration depth.

While the efficacy of PRF in expediting the healing process has been established, the latest systematic reviews indicate that there are no discernible supplementary advantages to be gained from using PRF in combination with bone materials during sinus floor elevation procedures [23, 24]. One of the primary advantages of implementing PRF in a crestal approach may be the mechanical protection of the sinus membrane during elevation, as well as the acceleration of the healing process in cases of micro‐perforation. In the context of an implant serving as a tent beneath the schneiderian membrane in a crestal approach, a sufficient amount of PRF around the implant after sinus floor elevation seems conducive to creating space for bone formation.

A scoping review conducted over the past 25 years, published in 2024, highlights that a key consideration across all minimally invasive techniques for transcrestal sinus augmentation is the protection of the sinus membrane from perforation [25]. According to this article, no clearly superior approach for sinus floor elevation using the transcrestal method can be identified due to the heterogeneity of the studies. Consequently, any method that minimizes the risk of sinus membrane perforation can be considered. For instance, techniques like the osteodensification method utilize a similar approach to protect the sinus membrane. What is proposed in the use of osseodensification system drills for close sinus floor elevation is also based on the pushing of bone debris resulting from the reverse rotation of the drills beneath the sinus membrane. During the creation of the osteotomy hole, the autogenous bone debris can act as a protective barrier for the sinus membrane. This may result in the membrane being moved away from the sinus floor bone, thereby minimizing the risk of any potential damage [26]. In the technique discussed in this article, PRF can serve a function analogous to that of autogenous bone debris within the osteodensification approach. Specifically, it is positioned between the drill and the sinus membrane.

According to a study conducted by Sonoda in 2017, to avoid sinus membrane perforation during the crestal approach, it is recommended that the ratio between the vertical elevation height of the membrane and the amount of membrane elevation in the mesiodistal and mediolateral dimensions should be ≤ 0.8 [27]. The findings of our study indicate that the observed changes in bone height are consistent with Sonoda's research findings.

Given the typically low bone density observed in the posterior maxilla, if sufficient initial stability for implant placement cannot be achieved, it is advisable to postpone the procedure until after appropriate repair and new bone formation occur. In such instances, it is recommended that following the transfer of PRF to the area between the Schneiderian membrane and the sinus floor bone, the osteotomy cavity be filled with bone powder. This technique helps to prevent the PRF from re‐entering the osteotomy cavity.

While there are notable advantages associated with the use of this modified technique, several concerns should be considered. A primary issue arises when the diameter and shape of the bone tap drill match those of the implant being utilized, which may lead to inadequate primary stability during implant insertion. To mitigate this risk, it is advisable to employ tapered implant drills when placing cylindrical implants in areas with lower bone density.

This study acknowledges several limitations, including a small sample size, the nature of the case report, and a relatively short follow‐up period of 1 year. While recognizing the limitations identified in this study, the methodology employed presents a promising approach for similar clinical situations. To thoroughly assess the long‐term efficacy and safety of this technique, it is advisable to conduct randomized controlled trials with larger sample sizes and extended follow‐up periods.

## Author Contributions

**Mehdi Ekhlasmandkermani:** conceptualization, investigation, methodology, supervision, visualization, writing – original draft, writing – review and editing. **Behzad Houshmand:** methodology, writing – original draft, writing – review and editing. **Emir Ilkerli:** methodology, writing – original draft, writing – review and editing. **Fatemeh Goudarzimoghaddam:** investigation, methodology, supervision, visualization, writing – original draft, writing – review and editing.

## Ethics Statement

The authors have nothing to report.

## Consent

Complete written informed consent was obtained from the patient for the publication of this study and the associated images.

## Conflicts of Interest

The authors declare no conflicts of interest.

## Data Availability

The data that support the findings of this study are available from the corresponding author upon reasonable request.
